# A practical framework for routine verification of Spot Position Monitor coordinate consistency in scanned carbon‐ion radiotherapy

**DOI:** 10.1002/acm2.70844

**Published:** 2026-10-05

**Authors:** Toshiro Tsubouchi, Noriaki Hamatani, Akira Komada, Masaaki Takashina

**Affiliations:** ^1^ Department of Radiation Oncology Piedmont Mountainside Hospital Jasper Georgia USA; ^2^ Department of Medical Physics Osaka Heavy Ion Therapy Center Osaka Japan

**Keywords:** carbon ion radiotherapy, log file‐based psQA, spot position monitor

## Abstract

**Background:**

Log file–based patient specific quality assurance (PSQA) in scanned particle therapy relies on the consistency of spot position information recorded by the spot position monitor (SPM), but practical methods for routine independent verification of these coordinates remain limited.

**Purpose:**

This study describes a practical framework for routine verification of SPM coordinate consistency using independent scintillator‐based measurements in raster‐scanning carbon‐ion radiotherapy.

**Methods:**

Reference spot positions were measured at the isocenter plane using a scintillator‐based detector. Corresponding SPM‐recorded spot coordinates were extracted from delivery log files. The two coordinate sets were aligned using a similarity transformation accounting for translation, rotation, and isotropic scaling. Post‐transformation root‐mean‐square (RMS) residuals were used as a practical consistency metric reflecting the agreement between SPM‐reported and independently measured spot positions under routine QA conditions.

**Results:**

After similarity transformation, the median RMS residual across all investigated accelerator energies and irradiation ports was 1.45 mm, with an interquartile range of 1.36–1.63 mm. No statistically significant difference was detected across accelerator energies, whereas a significant difference was observed among irradiation ports.

**Conclusions:**

The proposed framework provides a practical approach for routine verification of SPM coordinate consistency and for establishing facility‐specific baselines through longitudinal monitoring. The RMS residual should not be interpreted as the intrinsic positional error of the SPM alone, but to provide a framework for establishing a facility‐specific baseline for monitoring the agreement between SPM‐reported and independently measured spot coordinates under routine QA conditions.

## 1 INTRODUCTION

Advances in radiation delivery techniques have enabled increasingly precise treatments, underscoring the indispensable role of quality assurance (QA). From routine machine QA to patient‐specific QA (PSQA), each step is essential to ensure both the safety and effectiveness of radiotherapy.^1^ PSQA is performed to confirm that the planned dose distribution is accurately delivered and reproducibly realized.^2^ Traditionally, this has relied on measurement‐based approaches using detector systems. Although devices such as ArcCHECK provide quasi‐three‐dimensional dose verification through surface measurements and dose reconstruction, direct volumetric dose measurement remains challenging, particularly in particle therapy where dose deposition is strongly depth‐dependent.^3^, ^4^, ^5^, ^6^ Consequently, in particle therapy, clinical verification is often limited to measurements at only a small number of depth points.

Since the commencement of clinical operations, the increasing number of treated patients has rendered conventional measurement‐based PSQA increasingly labor‐intensive and time‐consuming, and insufficient for comprehensive verification of beam delivery under clinical conditions. This motivated the development and subsequent clinical implementation of a log file–based PSQA (LPSQA) system.^7^ In this framework, the spot positions recorded by the spot position monitor (SPM) and the delivered doses recorded by the dose monitor (DM) in the log file are combined with pre‐calculated single‐spot dose distributions in water, obtained using Monte Carlo simulations for each beam energy.^8^ The reconstructed dose distribution in water is then compared with the treatment plan to verify the accuracy of beam delivery. Only a single test irradiation prior to patient treatment is required to obtain the log file, eliminating the need for repeated detector measurements. This approach improves the efficiency of PSQA and enables three‐dimensional evaluation of the reconstructed dose distribution. In recent years, numerous studies have reported on LPSQA, demonstrating its potential for detailed analysis and improved QA accuracy.^7^, ^9^, ^10^, ^11^, ^12^ Log file–based QA has already been introduced into clinical practice and is considered particularly important in the context of adaptive radiotherapy.

On the other hand, log file–based QA relies on the consistency of delivery records, including spot position information recorded by the SPM integrated into the treatment nozzle. The SPM continuously monitors spot positions during delivery and is part of the beam delivery control system. Therefore, routine independent verification of SPM‐reported coordinates is useful for confirming the stability of the coordinate information used in log file–based PSQA. However, residual differences between SPM‐recorded and independently measured spot positions should be interpreted carefully, because they may include not only SPM‐related uncertainty but also measurement uncertainty, setup reproducibility, and coordinate registration uncertainty.

In this study, we describe a practical framework for routine verification of SPM coordinate consistency in raster‐scanning carbon‐ion radiotherapy. Independently measured spot positions acquired using a scintillator‐based detector were compared with SPM‐recorded coordinates extracted from delivery log files. A similarity transformation was applied to remove global coordinate differences between the measurement and SPM coordinate systems, and post‐transformation root‐mean‐square residuals were used as a practical constancy metric. The purpose of this work was not to isolate the intrinsic positional accuracy of the SPM alone, but to establish a facility‐specific baseline method for monitoring the agreement between SPM‐reported and independently measured spot coordinates under routine QA conditions.

## 2 MATERIALS AND METHODS

### 2.1 Machine specifications and periodic QA

The treatment system at a clinical center employs a raster‐scanning carbon‐ion beam delivery method.^13^ Clinically, 100 energies are available, achieved through a combination of 12 accelerator energies and range shifters, with a layer spacing of 3 mm in water‐equivalent thickness. The position and dose of each spot are continuously monitored by the SPM and the DM. Safety interlocks are incorporated to automatically terminate beam delivery if preset thresholds are exceeded. The SPM is integrated into the clinical beam delivery control system and it employs a sequential feedback mechanism in which positional deviations are accumulated over the initial spots and subsequently used to adjust the beam position of later spot deliveries. The present framework was designed to evaluate the consistency of recorded spot coordinates used in log file–based QA, rather than to isolate the intrinsic response characteristics of the SPM sensor or feedback algorithm alone. The facility comprises three treatment rooms, each equipped with two fixed beam ports. In treatment room FX1, the available ports are a sloped port and a horizontal port, whereas treatment rooms FX2 and FX3 are equipped with horizontal and vertical ports. Daily QA procedures include verification of absolute dose, range checks at selected energies, and confirmation of coincidence between the imaging isocenter and the beam axis, which is essential for image‐guided radiotherapy (IGRT). Monthly QA includes beam profile verifications such as flatness and symmetry, as well as verification of spot position accuracy across all 12 accelerator energies, which is performed in one treatment room per month on a rotating basis. These procedures represent standard QA practices in particle therapy facilities. The measurements analyzed in this study were acquired as part of the institution's routine monthly QA workflow.

### 2.2 Reference data acquisition using the XRV 2000 system

To obtain reference data for geometric alignment between SPM log coordinates and measured beam positions, a scintillator‐based beam profiler (XRV 2000 Falcon, Logos Systems Int'l, Scotts Valley, CA, USA) was placed at the isocenter plane. The system provides a 200 × 200 mm active scintillator area coupled with an optical imaging unit and dedicated analysis software, enabling beam centroid measurements with an accuracy of approximately ± 0.2 mm and a repeatability of ± 0.04 mm. The detector accuracy and centroid determination uncertainty may contribute to the residual differences evaluated in this study.

For each irradiation port and energy setting, nine reference spots—including the central axis and eight surrounding positions arranged in a 3 × 3 grid—were irradiated (Figure 1). The nominal maximum scanning field size was 20 × 20 cm^2^ for all irradiation ports. Although the active scintillator area was 200 × 200 mm^2^, the peripheral reference positions were limited to ± 8 cm from the central axis to minimize potential edge effects near the scintillator boundaries. The irradiation conditions are summarized in Table 1. Figure 2 illustrates the detector setup at the isocenter plane for each irradiation port. For each irradiation port, all 12 accelerator energies were measured without repositioning the XRV‐2000 detector, thereby maintaining the same measurement setup across energies. The nine‐point configuration was selected as a practical compromise between spatial sampling and routine QA feasibility. The pattern included the central axis and peripheral positions distributed over the clinically relevant irradiation field, allowing estimation of global translation, rotation, and scaling components while maintaining a measurement procedure compatible with routine monthly QA. The configuration was not intended to comprehensively characterize local nonlinear distortions over the entire field.

**FIGURE 1:**
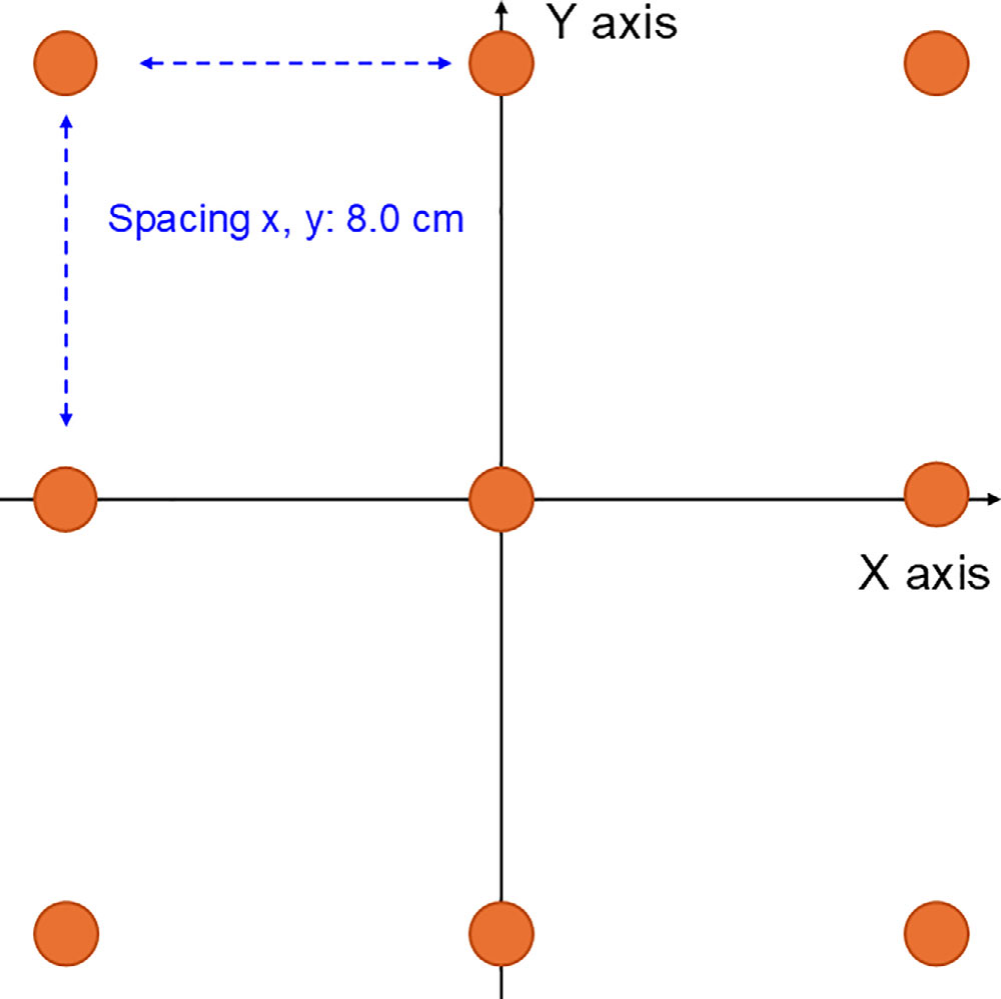
Configuration of reference spot positions used for SPM verification. Nine spots, consisting of the central axis and eight surrounding positions arranged in a 3 × 3 grid, were irradiated at the isocenter plane.

**TABLE 1:** Summary of irradiation and measurement conditions. The table lists the treatment rooms, irradiation ports, accelerator energies, and reference spot configuration employed in this study.

Room	3 rooms (FX1, FX2, and FX3)
Irradiation port	Vertical, horizontal, and slope ports
Maximum scanning field size [cm^2^]	20 × 20
Irradiation Energy [MeV/u]	100.0, 140.0, 170.9, 208.3, 241.9, 272.8, 302.1, 329.6, 356.1, 381.6, 406.2, 430.0
# of spots	9
Spot spacing (x and y) [cm]	8.0

**FIGURE 2:**
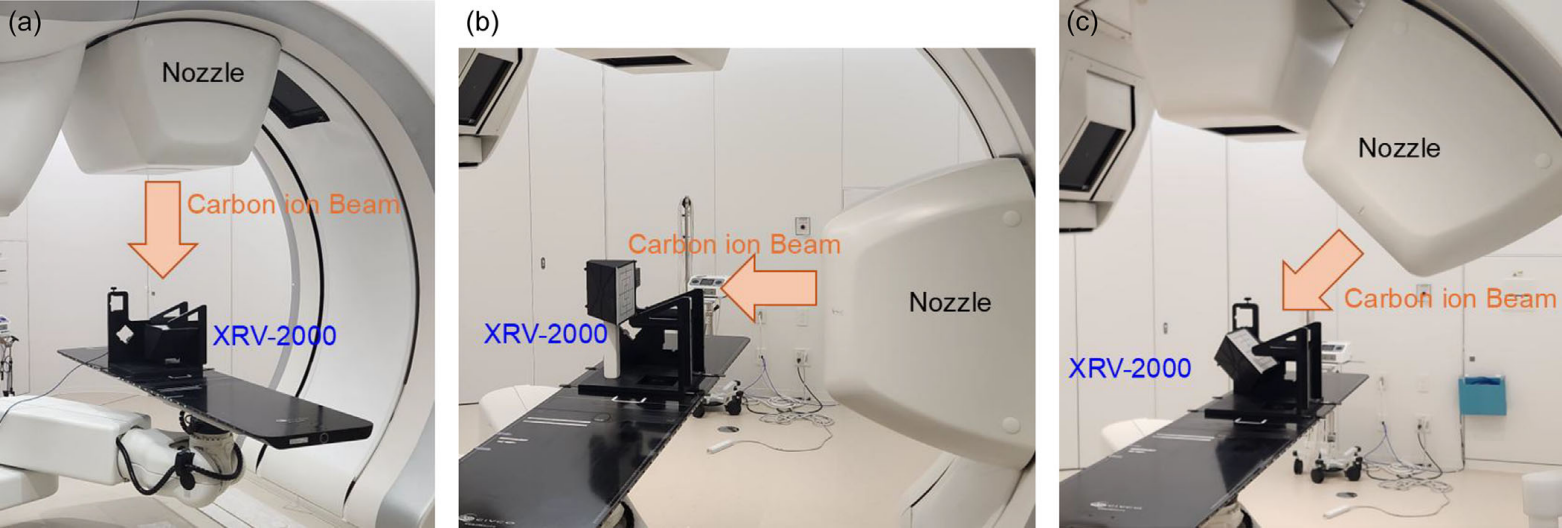
The detector setup at the isocenter plane for each irradiation port: (a) Vertical, (b) Horizontal, and (c) Slop (45 deg.) ports. The images illustrate consistent detector positioning (XRV‐2000) and measurement geometry across different beam delivery directions.

During irradiation, the XRV‐2000 system captured visible‐light images of the beam spots on the scintillator screen. The centroid positions of each spot were determined using an in‐house Gaussian‐fitting program to obtain measured coordinates $\left(x_{i}^{iso},\;y_{i}^{iso}\right)$. The corresponding log file entries were extracted to obtain SPM‐recorded coordinates $\left(x_{i}^{SPM},\;y_{i}^{SPM}\right)$. These paired coordinate sets were used to derive the similarity transformation parameters, as described in Section 2.3. The present study did not independently evaluate the influence of non‐Gaussian spot shapes or beam halo components on centroid estimation.

### 2.3 Similarity transformation and residual analysis

To align the SPM coordinate system with the measured coordinate system at the isocenter plane, a similarity transformation was applied. This transformation was used to account for global geometric differences between the SPM coordinate system and the independently measured coordinate system, including translation, rotation, and isotropic scaling differences arising from detector setup and coordinate registration. The similarity transformation can be expressed as:

$$\;\left(\begin{array}{c} x^{iso}\\ y^{iso} \end{array}\right)=\left(\begin{array}{c} s\cdot cos\left(\theta \right)\\ s\cdot sin\left(\theta \right) \end{array}\begin{array}{c} -s\cdot sin\left(\theta \right)\\ s\cdot cos\left(\theta \right) \end{array}\right)\;\left(\begin{array}{c} x^{SPM}\\ y^{SPM} \end{array}\right)+\left(\begin{array}{c} t_{x}\\ t_{y} \end{array}\right)$$

Here, s denotes the isotropic scaling factor, θ represents the rotation angle between the two coordinate systems, and $t_{x}$ and $t_{y}$ denote the translation components in the lateral directions. For each irradiation port, a single set of similarity transformation parameters was determined using all paired coordinates from the nine reference positions across the 12 accelerator energies by least‐squares fitting. The resulting port‐specific transformation was applied to all energy datasets acquired under the same measurement setup. For each energy, the residual error for each reference spot was calculated as the Euclidean distance between the transformed SPM coordinate and the corresponding measured coordinate, and the RMS of the nine residuals was used to quantify post‐registration coordinate agreement. In this study, the RMS residual was used as a practical coordinate consistency metric, rather than as a direct measure of intrinsic SPM positional accuracy. Because the residuals may include contributions from scintillator measurement uncertainty, centroid estimation uncertainty, beam delivery variation, and residual setup and coordinate registration uncertainties not accounted for by the global similarity transformation, the RMS residual should be interpreted as a practical constancy metric under routine QA conditions. Differences in RMS residuals across accelerator energies and irradiation ports were evaluated using the Friedman test to account for the repeated‐measures structure of the dataset. Irradiation ports were treated as blocks for the energy comparison, whereas accelerator energies were treated as blocks for the irradiation‐port comparison, with a significance level of *p* < 0.05.

## 3 RESULTS

### 3.1 Similarity transformation and post‐registration coordinate agreement

Figure 3 shows a representative example of the alignment between SPM‐recorded spot coordinates and independently measured reference coordinates after application of the similarity transformation. A single set of transformation parameters was obtained for each irradiation port using the measurements across all 12 accelerator energies, as summarized in Table 2.

**FIGURE 3:**
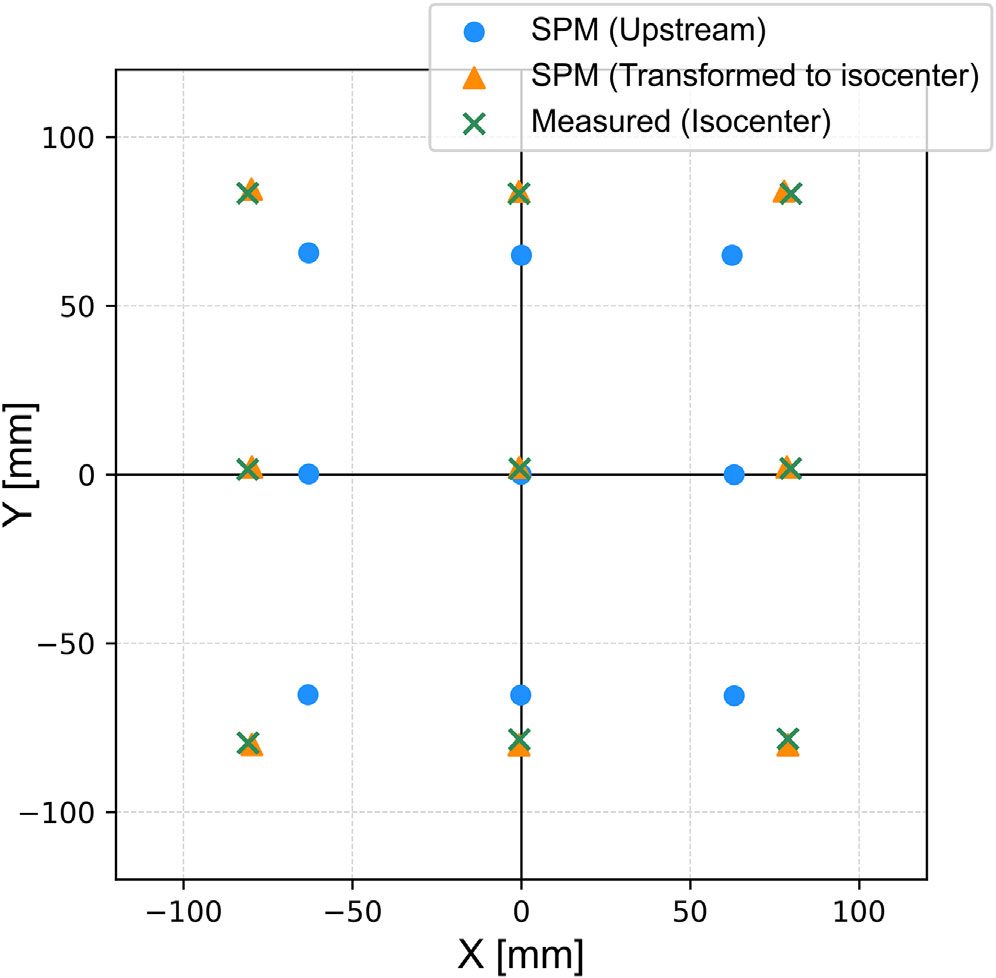
Example of spot position alignment achieved using the similarity transformation. SPM‐recorded spot positions are shown before and after transformation and compared with independently measured reference positions at the isocenter plane. The similarity transformation removes global geometric offsets, including translation, rotation, and isotropic scaling, resulting in close agreement between the transformed SPM positions and the reference measurements. The remaining spot‐by‐spot deviations represent post‐registration coordinate differences between the transformed SPM positions and the reference measurements.

**TABLE 2:** Similarity transformation parameters for each irradiation port.

	Scale factor, s	Rotation angle, θ [deg]	$t_{x}$ [mm]	$t_{y}$ [mm]
FX1H	1.25	−0.08	0.19	−0.06
FX1S	1.25	−0.04	−0.18	−0.15
FX2H	1.25	0.05	0.30	−0.11
FX2V	1.25	−0.04	−0.14	−0.32
FX3H	1.25	−0.09	0.04	0.10
FX3V	1.25	0.00	−0.10	−0.16

### 3.2 RMS residuals across energies and irradiation ports

RMS residuals were calculated for 72 energy‐port combinations (12 accelerator energies × 6 irradiation ports). Figure 4a shows the distributions of RMS residuals for each accelerator energy, and Figure 4b shows the corresponding distributions for each irradiation port.

**FIGURE 4:**
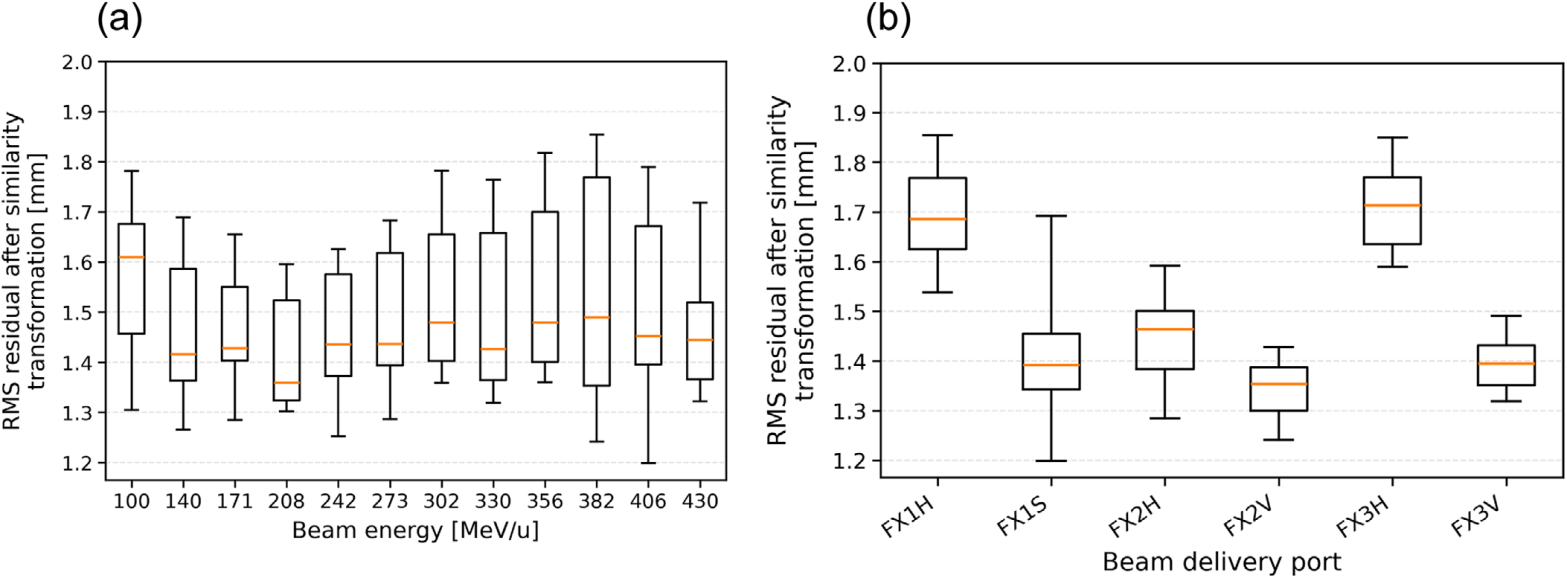
Distributions of RMS residuals after similarity transformation. (a) Energy‐dependent RMS residuals across all accelerator energies. (b) Port‐dependent RMS residuals for different irradiation ports. Ports are labeled by room and beam direction, for example, FX1H and FX3V, where “FX1/FX3” indicate treatment rooms and “H”, “V”, and “S” denote horizontal, vertical, and sloped ports, respectively.

Across all evaluated energies and irradiation ports, the median RMS residual was 1.45 mm, with an interquartile range of 1.36–1.63 mm. No statistically significant difference in RMS residuals was observed across accelerator energies (Friedman test, χ^2^(11) = 13.87, *p* = 0.240). In contrast, a statistically significant difference was observed among irradiation ports (χ^2^(5) = 41.76, *p* < 0.001).

## 4 DISCUSSION

In this work, we described a practical framework for routine verification of coordinate consistency between SPM‐reported spot positions and independently measured positions in raster‐scanning carbon‐ion radiotherapy. Unlike conventional spot‐position QA, which primarily verifies the delivered beam position against predefined reference positions, the present framework specifically evaluates the coordinate information recorded by the SPM and subsequently used in log file–based PSQA. Thus, the method provides an independent check of the coordinate data underlying log file–based dose reconstruction. The primary purpose of this approach is to provide a framework for establishing a facility‐specific baseline for post‐registration coordinate agreement under routine QA conditions.

A key point in the interpretation of this framework is that the RMS residual after similarity transformation should not be regarded as a direct measure of the intrinsic positional accuracy of the SPM alone. The similarity transformation accounts for global coordinate differences between the SPM and scintillator measurement systems, including translation, rotation, and isotropic scaling. Therefore, the residual RMS primarily reflects the remaining post‐registration coordinate disagreement after these global components have been removed. Accordingly, absolute global geometric offsets are not directly quantified by this RMS metric. The residual may include contributions from SPM‐reported coordinate uncertainty, scintillator measurement uncertainty, centroid estimation uncertainty, beam delivery variation, and residual setup or coordinate registration effects not accounted for by the global similarity transformation. These contributions cannot be independently separated using the present measurement framework. Consequently, an increase in RMS residual during longitudinal monitoring would indicate a change in overall coordinate consistency but would not, by itself, identify the underlying source. For this reason, the RMS residual is best interpreted as a practical coordinate consistency or constancy metric under routine QA conditions.

The median RMS residual observed in this study was 1.45 mm, with an interquartile range of 1.36–1.63 mm across the evaluated accelerator energies and irradiation ports. No statistically significant difference in RMS residuals was detected across accelerator energies, whereas a significant difference was observed among irradiation ports. The observed port dependence should not be interpreted as evidence of differences in intrinsic SPM positional accuracy because the RMS residual reflects combined contributions from the measurement and delivery chain. Rather, the results suggest that systematic characteristics associated with individual irradiation‐port measurement setups may influence the observed RMS residuals. These findings should be considered when establishing port‐specific baseline values for future longitudinal monitoring.

For practical implementation, an initial port specific baseline could be established from repeated measurements acquired under stable operating conditions during routine monthly QA. The central tendency and variability of the resulting RMS residuals could then be used to define local warning or investigation levels for longitudinal monitoring. Because longitudinal data were not available in the present study, specific numerical control limits cannot be determined from the current dataset. Such limits should therefore be established prospectively based on institutional measurement variability and refined as additional longitudinal data become available.

The nine point reference pattern was selected as a practical compromise between spatial sampling and routine QA feasibility. The configuration included the central axis and peripheral positions, allowing estimation of global translation, rotation, and scaling components while maintaining a measurement procedure compatible with routine monthly QA. However, the pattern was not designed to comprehensively characterize local nonlinear geometric distortions over the entire irradiation field. Rather, its purpose was to provide a reproducible sampling pattern for routine constancy monitoring using a global similarity transformation. A denser sampling pattern or higher‐order spatial transformation would be required if detailed characterization of local field‐dependent distortions or absolute spatial calibration were the objective.

Several limitations should be noted. First, this study did not include longitudinal trend data. Therefore, although the proposed framework is intended for future trend monitoring, the ability of this method to detect gradual changes in coordinate consistency or abrupt changes in system performance was not directly demonstrated. Second, the analysis was performed using controlled reference spot patterns rather than patient‐specific spot distributions. The present work therefore does not evaluate the behavior of the framework for complex clinical spot patterns. Third, the dosimetric consequences of the observed residual differences were not assessed. The dosimetric impact of a given coordinate residual may depend on factors such as the spatial pattern of spot deviations, spot spacing, beam size, and local dose gradients, and therefore cannot be inferred directly from the RMS coordinate metric alone. Further studies would be required to determine how changes in this coordinate consistency metric propagate into reconstructed dose distributions or clinical PSQA decision metrics.

The influence of non‐Gaussian spot profiles or beam halo components on centroid estimation was not independently evaluated. This limitation should also be considered when interpreting the RMS residuals.

The framework is facility‐specific in its implementation because the observed RMS residuals may depend on the delivery system, SPM characteristics, detector system, measurement setup, and coordinate registration method. Therefore, universal tolerance values should not be inferred from the present results. Nevertheless, the conceptual approach may be transferable to other raster‐scanning particle therapy systems, provided that each institution establishes its own baseline and interprets residual changes in the context of its own measurement uncertainty and QA workflow.

## 5 CONCLUSION

The proposed framework provides a practical method for routine verification of SPM coordinate consistency using independent scintillator measurements and delivery log data. Rather than directly quantifying intrinsic SPM accuracy, the method provides a framework for establishing a facility‐specific baseline for post‐registration coordinate agreement and may support future trend‐based monitoring of SPM‐reported coordinate consistency within log file–based PSQA workflows.

## AUTHOR CONTRIBUTIONS

**Toshiro Tsubouchi**: Conceptualization; methodology; formal analysis; data acquisition and analysis; data curation; writing—original draft preparation; writing—review and editing; visualization; project administration. **Noriaki Hamatani**: Conceptualization; methodology; writing—review and editing. **Akira Komada**: Data acquisition and analysis; data curation. **Masaaki Takashina**: Conceptualization; methodology; supervision. All authors critically revised the report, commented on drafts of the manuscript and approved the final report.

## CONFLICT OF INTEREST STATEMENT

The authors have no conflicts to disclose.

## ETHICS STATEMENT

This study did not involve human participants, patient data, or animals; therefore, institutional review board approval was not required.

## GENERATIVE AI STATEMENT

During the preparation of this manuscript, the author(s) used ChatGPT (Model: GPT‐4o, OpenAI) to improve the grammatical structure, readability, and English phrasing of the text. After using this tool, the author(s) reviewed and edited the content as needed and take(s) full responsibility for the content of the publication.

## Data Availability

The data that support the findings of this study are available from the corresponding author upon reasonable request.
